# Gender, nutritional status and disability-free life expectancy among older people in Santiago, Chile

**DOI:** 10.1371/journal.pone.0194074

**Published:** 2018-03-28

**Authors:** Ximena Moreno, Cecilia Albala, Lydia Lera, Bárbara Leyton, Bárbara Angel, Hugo Sánchez

**Affiliations:** Institute of Nutrition and Food Technology, University of Chile. ElLíbano, Macul, Santiago, Chile; Scientific Institute of Public Health (WIV-ISP), BELGIUM

## Abstract

**Background:**

This study was aimed to estimate life expectancy (LE), disability-free life expectancy (DFLE) and disabled life expectancy (DLE) among older adults from Santiago, Chile, and to determine the existence of differences by gender and by body mass index (BMI) categories in these indicators.

**Methods:**

A sample of 1216 people aged 60 or more, from the Chilean cohort of the Study of Health, Ageing and Well-Being was recruited in 2000; two follow-up assessments were carried out in a 10-year period. Functional limitation was assessed through self-report of difficulties in activities of daily living, instrumental activities of daily living and mobility. BMI was determined with measured weight and height. Multistate life tables were employed to estimate LE and healthy life expectancy (HLE).

**Results:**

At 60 years, women could expect to live on average an additional 20.4 years (95% CI 19.0–21.6), and men an additional 16.4 years (95% CI 14.9–17.7). Total LE was longer among women at all ages, but they had a higher proportion of disabled years to be lived compared to men, with a difference of 14% at 60 years, and 10% at 90 years. There were no significant differences in LE, DFLE and DLE between BMI categories.

**Discussion:**

Despite a longer LE, Chilean older women expect to live a higher proportion of years with disabilities, compared to men. Public health programs should address factors affecting LE of older men, and those associated with disability among older women.

## Background

The ageing process exhibits particular features in Latin America and the Caribbean. As in many developing countries, a rapid transition from a young to an old age structure is occurring; however, unlike developed countries, this process is occurring at a high speed, with much less time to achieve the economic development needed to cover the demands of older adults on financial security, social protection, environmental support and health care [1]. There is considerable heterogeneity with respect to life expectancies (LE) in the region. In some countries LE at birth and at 60 years are comparable to European countries. The most remarkable case is Chile, where, in 2015, LE at birth was 80.5, and LE at 60 years was 24.4 [2]. Considering that older people in Chile can expect to live as long as older people from countries where the ageing process started earlier and developed at a slower pace, questions with respect to the quality of those years to be lived are raised.

Health expectancies are an important tool to assess the quantity and quality of years to be lived by specific groups of the population [3], allowing us to combine information on survival and health, instead of considering them in isolation [4].Previous research on disability-free life expectancy (DFLE) has explored the gender health-survival paradox, consisting of a mortality advantage and disability disadvantage of women [5]. Research has shown that the gender gap varies, depending on structural indicators such as health expenditure or poverty risk of the country [6]. Factors related to lifestyle have also been proposed as a mechanism behind the gender gap in health expectancies, due to their differential distribution among men and women and their impact on morbidity and mortality [5,7]. Obesity is considered a risk factor for morbidity and mortality among the general population [8–10]. Numerous studies have reported that obesity increases the risk of developing disability [11–14]. Studies on the impact of obesity on LE and DFLE in older people, carried out in Europe and North America, have shown no impact of obesity on LE of older people, compared to those with a normal body mass index (BMI) [15–20], and a higher proportion of years expected to be lived with disabilities [15–18,21]. Similarly, two studies found that overweight women had a longer DLE [17,22], and two reported a longer LE among overweight participants [21,22].

Obesity and overweight are common among older people in Chile. In 2010, 42.6% of people aged 65 or more were overweight, and 31% were obese [23]; a recent Chilean study found that 40% of women and 28% of men aged 60 or more are obese [24]. The scale of the gender gap in LE and DFLE of Chilean elders, is unknown to date, and the impact of obesity and overweight on these indicators has not been studied. This study will determine if there is a difference in DFLE and DLE between older men and women and by BMI category in Santiago, Chile.

## Methods

We used data from the Health, Well-being and Ageing (SABE) study, carried out in seven large cities of Latin America and the Caribbean. Methodological details of this study have been described before [25–26]. The Chilean sample consisted of 1301 individuals living in the community in Santiago, born before 1940 and recruited in 1999–2000 through a probabilistic sampling process with a three-step procedure, considering districts, blocks and households. The results reported here are based on 1216 individuals who had complete baseline data on weight and height, in order to estimate their BMI. Excluded individuals had a higher prevalence of limitations in two or more instrumental activities of daily living, compared to those included in the analyses (32.5% vs. 18.7%, p = 0.002). No other statistically significant difference on baseline socio-demographic characteristics or health status between both groups was observed.

The baseline assessment consisted of a home interview, carried out by trained interviewers and included weight and height measurements and questions on health and functional status. Two follow-up measurements were carried out in 2004–2005 [27] and in 2009–2010 [28], including questions about functional status of participants. As observed in S1 Fig, by the second interview, 225 participants had died, and 577 were interviewed. Of 991 participants still alive after the second assessment, 351 were interviewed, 236 had died and 404 were alive but their health status was unknown, at the end of the study follow-up. Participants who only had baseline information were younger than those who were followed-up (p<0.001), and had a higher prevalence of limitation in one or more activities of daily living (ADL) (p = 0.01) and in two or more instrumental activities of daily living (IADL) (p = 0.04), but the distribution by sex, BMI and functional status was similar between both groups.

Functional status was determined by self-report of the ability to perform ADL -including bathing, dressing, toileting, transferring, feeding and walking-; IADL -considering ability to handle finances, taking own medications, cooking, housekeeping and making phone calls-; and mobility function (MF)–assessed through the following activities and actions: vigorous activities such as lifting heavy objects, moderate activities such as sweeping, lifting groceries, climbing one set of stairs, climbing several sets of stairs, bending/kneeling/stooping, walking one block, walking several blocks, walking more than a mile, pulling or pushing large objects, lifting or carrying weights over 10 pounds, and extending arms above shoulder level. We defined functional limitation according to the criteria that Albala et al. [29] propose for the Chilean older population, namely limitation in at least one ADL, in two IADL, or in three MF questions. Weight and height measured at baseline were employed to determine BMI, using World Health Organization (WHO) BMI cut-offs points [30]: less than 18.5 as underweight, 18.5 to 24.9 as normal, 25 to 29.9 as overweight, and 30 or more as obesity. The vital status of all participants was determined at the end of follow-up. Mortality was ascertained through death certificates up to the end of 2010, which were obtained from the National Civil Registration Office.

To estimate LE, DFLE and DLE, we employed a multistate life tables approach. We used the Multi-State Markov and Hidden Markov Models in Continuous Time package (msm, version 1.6.4) for R (version 3.4.2), to estimate the transition intensity matrix [31]. With this package, a model can be fitted to longitudinal data with a mixture of arbitrary observation times, exact death times and censored states, and the effect of covariates can be explored [32]. LEs were estimated from the multi-state model with the “Estimation of Life Expectancies Using Continuous-Time Multi-State Survival Models” functions (ELECT, version 1.03) for R. A three-state model was fitted: state 1 was “healthy” (without functional decline), state 2 was “unhealthy” (with functional limitation), and state 3 was “dead”. Four transitions were possible: health to disability onset, disability to health, health to death and disability to death. Participants who were alive but in an unknown health status, by the end of follow-up, were right-censored. The multi-state models included age as a time-dependent variable, and sex and baseline BMI as covariates. LEs were estimated by gender and by BMI and gender; 95% confidence intervals were computed by simulation (1000 repetitions). Due to the low prevalence of underweight (1.6%), LEs for this category were not estimated.

This study was approved by the ethics committees of the Pan-American Health Organization and the Institute of Nutrition and Food Technology of the University of Chile. All participants signed an informed consent to take part in the study.

## Results

Baseline age, nutritional status according to BMI and functional status of the sample are depicted in Table 1. The average age was 71.5 and ranged from 60 to 99. Among men and women, the most frequent BMI category was overweight, but obesity was more frequent among women. Baseline functional limitation was more frequent in women, who had a more than double prevalence of difficulties in IADL and mobility, compared to men.

**Table 1:** Baseline characteristics of the sample .

	Men	Women	p
	n = 410	n = 806	
Age (mean, s.d.)	70.6 (7.4)	71.9 (8.2)	0.004
60–69 years (%)	56.6	49.3	0.003
70–79 years (%)	32.9	33.2	
80 or more (%)	10.5	17.5	
Nutritional status (BMI)			
Underweight (%)	1.5	1.7	0.002
Normal (%)	30.5	25.3	
Overweight (%)	45.1	39.3	
Obese (%)	22.9	33.6	
Limitation in one or more ADL (%)	17.1	28.7	<0.001
Limitation in two or more IADL (%)	10.0	23.1	<0.001
Limitation in three or more MF (%)	18.1	43	<0.001
Functional limitation (%)	29.8	54.2	<0.001

Table 2 shows the Hazard Ratio of specific transitions, by age, gender and BMI. One additional year of age increased the risk of disability incidence (HR = 1.06, 95% CI 1.04–1.09) and mortality among disabled individuals (HR = 1.06, 95% CI 1.04–1.08). Women were more likely to become disabled (HR = 1.82, 95% CI 1.30–2.58) and less likely to die from the non-disabled state (HR = 0.15, 95%CI 0.02–0.90) and from the disabled state (HR = 0.64, 95% CI 0.49–0.83), compared to men.

**Table 2:** Hazard ratios of disability incidence, recovery and mortality from specific health state, by age, gender and BMI (n = 1196).

	Age		Female		Overweight		Obese	
			(Ref.: Male)		(Ref.: Normal)		(Ref.: Normal)	
	HR	95% CI	HR	95% CI	HR	95% CI	HR	95% CI
Disability incidence	1.06	1.04–1.09	1.82	1.30–2.58	1.03	0.69–1.55	1.56	0,96–2.54
Recovery	0.97	0.94–1.10	1.37	0.79–2.38	0.74	0.38–1.50	1.42	0.68–2.95
Mortality of non-disabled	1.06	0.99–1.12	0.15	0.02–0.90	0.93	0.40–2.20	0.10	0.01–1.60
Mortality of disabled	1.06	1.04–1.08	0.64	0.49–0.83	0.81	0.61–1.10	0.91	0.67–1.22

Hazard ratios from a multistate Markov model with continuous time, including age, sex and body mass index as covariates.

Ref.: Reference.

**Fig 1:**
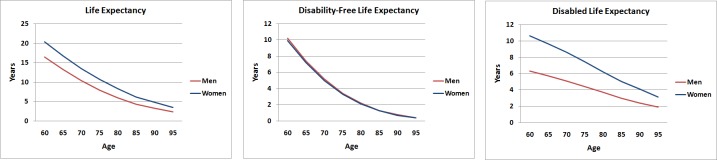
Life expectancy, disability-free life expectancy and disabled life expectancy in men and women.

As observed in Table 3, women had a longer LE at all ages compared to men. On average women at ages 60 and 70 could expect to live an additional 20.4 and 13.5 years, respectively. At ages 60 and 70, men could expect to live an additional 16.4 and 10.3 years, respectively. However, gender differences in the proportion of years to be lived with disabilities favoured men until very old ages (Fig 1). This difference was higher than 10% until 90 years of age. Differences in years expected to be lived free of disability were not statistically significant for any age. Table 4 shows LE, DFLE and DLE by nutritional status. In this case, no statistically significant differences in LE, DFLE and DLE by BMI category were observed.

**Table 3:** Total life expectancy (LE), disability-free life expectancy (DFLE), disabled life expectancy (DLE), and percentage of years to be lived with disability (%disabled), for men and women (n = 1196).

	Age 60		Age 70		Age 80		Age 90	
	Male	Female	Male	Female	Male	Female	Male	Female
Total LE	16.4	20.4	10.3	13.5	5.9	8.3	3.2	4.8
95% CI	14.9–17.7	19.0–21.6	9.4–11.3	12.5–14.5	5.1–6.7	7.5–9.2	2.5–3.9	4.0–5.6
DFLE	10.2	9.9	5.2	4.9	2.2	2.1	0.8	0.1
95% CI	9.0–11.4	8.8–10.8	4.4–6.0	4.3–5.6	1.7–2.8	1.7–2.6	0.5–1.1	0.5–1.0
DLE	6.3	10.6	5.1	8.6	3.7	6.2	2.4	4.1
95% CI	5.2–7.2	9.4–11.6	4.4–5.9	7.6–9.5	3.0–4.5	5.4–7.1	1.8–3.1	4.0–5.6
% Disabled	38.4	52.0	49.5	63.7	62.7	74.7	75.0	85.4

**Table 4:** Total life expectancy (LE), disability-free life expectancy (DLFE), disabled life expectancy (DLE), and percentage of years to be lived with disability (% disabled), by nutritional status, for men and women (n = 1196).

	BMI	LE	95% CI	DFLE	95% CI	DLE	95% CI	% Disabled
Men								
Age 60	Normal	15.5	12.8–18.1	9.3	6.5–12.5	6.2	4.1–8.6	40
	Overweight	15.5	12.6–17.8	10.4	8.1–10.4	5.1	3.5–6.7	32.9
	Obese	16.5	0.2–22.6	10.3	0–14.5	6.3	0.1–10.3	38.2
Age 70	Normal	10.1	8.5–12.1	4.9	3.3–7.2	5.2	3.4–7.3	51.5
	Overweight	10.3	8.7–12.1	5.9	4.6–7.5	4.4	3.2–5.9	42.7
	Obese	11.2	3.5–15.4	5.1	1.0–8.2	6.1	2.4–9.4	54.5
Age 80	Normal	6.5	4.9–8.5	2.5	1.4–4.7	4.0	2.0–5.9	61.5
	Overweight	6.2	4.6–8.4	2.8	1.8–4.5	3.4	2.0–5.2	54.8
	Obese	7.2	3.6–11.6	2.3	0.6–4.8	4.9	2.4–8.9	68.1
Age 90	Normal	4.0	2.5–6.1	1.1	0.4–2.8	2.9	1.1–5.0	72.5
	Overweight	3.5	2.0–5.6	1.1	0.4–2.6	2.4	1.0–4.5	68.6
	Obese	4.3	1.4–8.2	0.8	0–2.9	3.4	1.0–7.3	79.1
Women								
Age 60	Normal	19.0	12.9–21.8	10.8	7.1–13.4	8.2	5.2–10.2	43.2
	Overweight	20.9	18.1–22.9	9.2	7.4–10.8	11.7	9.7–13.6	56.0
	Obese	19.5	12.7–22.1	9.0	5.9–10.7	10.5	6.6–12.8	53.8
Age 70	Normal	12.7	10.8–14.4	5.8	4.5–7.2	6.9	5.5–8.4	54.3
	Overweight	14.2	11.9–16.1	5.0	3.9–6.3	9.2	7.4–10.9	64.8
	Obese	13.1	10.9–15.1	4.1	3.1–5.5	9.0	7.1–10.8	68.7
Age 80	Normal	7.4	5.7–8.7	2.5	1.6–3.5	4.9	3.7–6.2	66.2
	Overweight	8.6	6.3–10.5	2.3	1.4–3.5	6.3	4.6–8.0	73.3
	Obese	8.4	6.4–10.9	1.6	0.9–2.7	6.8	4.8–9.2	81.0
Age 90	Normal	4.0	2.7–5.4	1.0	0.4–1.8	3.0	2.0–4.4	75.0
	Overweight	4.7	2.9–6.8	1.0	0.3–2.1	3.8	2.2–5.6	80.9
	Obese	5.1	3.2–7.2	0.6	0.1–1.4	4.5	2.7–6.7	88.2

## Discussion

The goal of this study was to determine the existence of a gender gap on DFLE and DLE of Chilean older men and women, and to explore the association of obesity and overweight with DFLE and DLE. As expected, we found that total LE was longer among women, but that they also could expect to live a higher proportion of their remaining years of life with disabilities. With respect to nutritional status, no significant differences were found between BMI categories. Our results are in line with a significant number of studies in high income countries and in developing economies, which have shown a longer LE and a lower proportion of DFLE among women [4,5,7,33–39]. Previous studies suggest that a lower level of education increases the risk of disability, even at very old ages [40], and that women are more likely than men to have low levels of education [41–43]. In our own study, women were less educated (p<0.001), with an average of 5.2 years of education, compared to 6.5 years of education among men. Other mechanisms that could explain the higher burden of disability among older women include a higher prevalence of underlying conditions such as musculoskeletal disorders and fall-related fractures [44–47]. Consistent with this, the prevalence of osteoarthritis among Chilean older women has been estimated to exceed 40%, which is more than double that of men, independent of socioeconomic position [28]. Similarly, the prevalence of osteoporosis among women in our study was more than 5 times higher than among men (15.5% vs. 2.2%, p<0.001) and women also more frequently reported falls during the last year compared to men (39.7% vs. 22.7%, p<0.001).

We studied overweight and obesity as defined by BMI, considered as lifestyle factors that could have an effect on the gender health-survival paradox. Previous studies have found that overweight and obese older adults can expect to live more of their years with disabilities [15–18,21]. In these studies, a longer LE has been observed only among overweight elders [21,22], with no effect of obesity on this indicator. Our results provided no evidence to support either a negative or a positive effect of overweight or obesity on LE and DFLE. Although obese individuals born before 1940 in our cohort did not have a higher proportion of disabled life years, compared to non-obese participants, this situation might be different for those born after 1940. A recent study found that cohorts of older adults born after 1940 could be more vulnerable to sarcopenic obesity, due to the important changes in lifestyle during the past three decades [24]. Elevated energy intake and physical inactivity are among the factors that increase the risk of developing this condition [48].

Our research has a number of strengths. First, our study is among the few published reports of DFLE estimates, from a prospective study, in older people in Latin America [49–52]. Secondly, in our study BMI was assessed by trained interviewers with standardized methods and equipment, minimizing measurement error. Furthermore, the sample was representative of older people living in the community in Santiago, the capital of Chile, and the methodological approach takes into account health transitions in our estimations. On the other hand, it is also important to consider some limitations. Although the sampling strategy allows us to extrapolate our results to the entire older population of the capital city, this is not the case for the rural population of Chile, where worse average living conditions and worse perceptions of health and quality of life exist, according to the National Study of Dependence among Older Adults [53]. Additionally, the sample size affected the precision of some estimations when stratified by BMI, particularly among obese men. These results should therefore be interpreted with caution. The use of BMI as a fixed covariate is a limitation of our analysis. Our approach is similar to most previous studies [15–22], and our assumption was that baseline BMI is associated to LE and DFLE. Nevertheless, our estimations are not expressing the effect of changes in BMI on LE and DFLE. It is important to assess the impact of BMI trajectories on LE and DFLE among older people, considering the evidence that suggests that changes in BMI increase the risk of disability and mortality [38]. Finally, due to the lack of retrospective data, it was not possible to rule out that previous obesity could have negatively affected the health status of individuals who, due to these health problems, experienced a weight loss and were not obese at baseline. This could have biased the estimation of the impact of obesity on LE and DFLE. Also, we could not monitor the incidence of potentially disabling conditions, in order to estimate their distribution across BMI categories and to consider their effect on our estimations.

Future research on this topic could be strengthened by collecting and analyzing information on past and future weight. According to previous research [54], overweight and obesity during the life course have an impact on the risk of physical disability at old age. Other lifestyle factors that could have an effect on LE and DFLE should also be considered, such as smoking and exercise. Finally, weight loss and weight cycling increase the risk of disability and mortality among older people [55], and weight loss among obese older people is associated with incident multimorbidity [56].

In conclusion, this is the first study to report estimates of DFLE and DLE among Chilean older people. According to our results, in 2000, older women from Santiago had a longer LE, but could expect to live a higher proportion of these additional years with disabilities, when compared to men. Gender health inequalities should be addressed by public health programmes, considering factors affecting LE among older men, and those increasing the risk of disability among older women.

## Supporting information

S1 Fig Number of participants interviewed and vital status on each stage. (TIF)

## Abbreviations

LE: Life Expectancies
DFLE: Disability-Free Life Expectancies
DLE: Disabled Life Expectancies
SABE: Study on Health, Well-being and Ageing
BMI: Body Mass Index
ADL: Activities of Daily Living
IADL: Instrumental Activities of Daily Living
MF: Mobility Function
