# Supplementary figures and images for: Gender, nutritional status and disability-free life expectancy among older people in Santiago, Chile
Source: PLoS One. 2018 Mar 28;13(3):e0194074. doi: 10.1371/journal.pone.0194074 (PMC5874002; doi:10.1371/journal.pone.0194074)

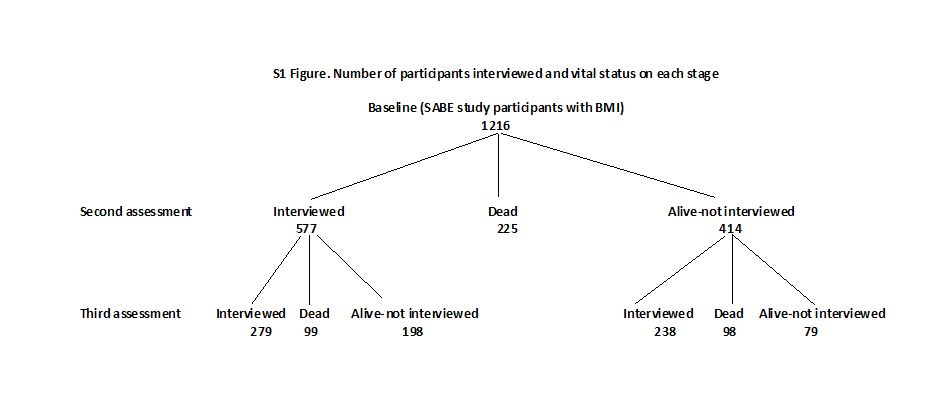

Supplement: S1 Fig — (TIF) [file pone.0194074.s001.tif]
